# Machine Learning-Based Integration of Metabolomics Characterisation Predicts Progression of Myopic Retinopathy in Children and Adolescents

**DOI:** 10.3390/metabo13020301

**Published:** 2023-02-17

**Authors:** Xiao-Wen Hou, Jin-Liu-Xing Yang, Dan-Lin Li, Yi-Jin Tao, Chao-Fu Ke, Bo Zhang, Shang Liu, Tian-Yu Cheng, Tian-Xiao Wang, Xun Xu, Xian-Gui He, Chen-Wei Pan

**Affiliations:** 1 School of Public Health, Medical College of Soochow University, Suzhou 215123, China; 2 Shanghai Eye Disease Prevention and Treatment Center, Shanghai Eye Hospital, Shanghai Vision Health Center & Shanghai Children Myopia Institute, Shanghai 200040, China; 3 Department of Ophthalmology, the First Affiliated Hospital of Kunming Medical University, Kunming 650032, China; 4 Department of Ophthalmology, Shanghai General Hospital, Shanghai Jiao Tong University, National Clinical Research Center for Eye Diseases, Center of Eye Shanghai Key Laboratory of Ocular Fundus Diseases, Shanghai Engineering Center for Visual Science and Photomedicine, Shanghai 200080, China

**Keywords:** myopic retinopathy, children and adolescents, metabolomics, machine learning, prediction model

## Abstract

Myopic retinopathy is an important cause of irreversible vision loss and blindness. As metabolomics has recently been successfully applied in myopia research, this study sought to characterize the serum metabolic profile of myopic retinopathy in children and adolescents (4–18 years) and to develop a diagnostic model that combines clinical and metabolic features. We selected clinical and serum metabolic data from children and adolescents at different time points as the training set (*n* = 516) and the validation set (*n* = 60). All participants underwent an ophthalmologic examination. Untargeted metabolomics analysis of serum was performed. Three machine learning (ML) models were trained by combining metabolic features and conventional clinical factors that were screened for significance in discrimination. The better-performing model was validated in an independent point-in-time cohort and risk nomograms were developed. Retinopathy was present in 34.2% of participants (*n* = 185) in the training set, including 109 (28.61%) with mild to moderate myopia. A total of 27 metabolites showed significant variation between groups. After combining Lasso and random forest (RF), 12 modelled metabolites (mainly those involved in energy metabolism) were screened. Both the logistic regression and extreme Gradient Boosting (XGBoost) algorithms showed good discriminatory ability. In the time-validation cohort, logistic regression (AUC 0.842, 95% CI 0.724–0.96) and XGBoost (AUC 0.897, 95% CI 0.807–0.986) also showed good prediction accuracy and had well-fitted calibration curves. Three clinical characteristic coefficients remained significant in the multivariate joint model (*p* < 0.05), as did 8/12 metabolic characteristic coefficients. Myopic retinopathy may have abnormal energy metabolism. Machine learning models based on metabolic profiles and clinical data demonstrate good predictive performance and facilitate the development of individual interventions for myopia in children and adolescents.

## 1. Introduction

In East Asian countries, 80% of high school graduates have myopia and 10% have high myopia (HM) [1,2]. The risk of myopic macular degeneration increases disproportionately among children with early-onset HM [3], suggesting that the risk of myopic retinopathy in children and adolescents needs to be taken seriously as the age of myopia onset advances and the number of cases continues to increase. In previous studies, fundus tessellation (FT) was found to be a good predictor of myopic retinopathy onset in adults with myopia [4], where FT development as diffuse atrophy is a common pattern of myopia progression [5]. The International Photographic Classification System is the primary standard for evaluating fundus lesions, but its clinical application is limited by intra- and interobserver variability, as well as by the workload of reading films and, most importantly, as well as by the workload of reviewing films, and most importantly, the standard was developed based on adult study subjects and may be too harsh for monitoring retinal damage in children and adolescents [6]. Previous studies of myopia risk factors have neglected the key group of children and adolescents, and few studies have addressed the early stages of retinopathy that impair vision [7,8,9,10]. There is no cure for myopic retinopathy, and early identification and monitoring would be a prudent strategy for myopia care in children and adolescents.

Previous studies have shown that metabolites with pleiotropic and biological properties can be detected in blood and that they may be involved in the development and progression of various intraocular diseases [11,12,13]. A review of myopia metabolomics found that population studies mainly used serum as biological samples (sample size 38–211) and included three types of studies: comparison of HM and nonmyopic controls, comparison of HM and mild myopia, and comparison of myopic and nonmyopic. The predictive value of biomarker panels shows the value of metabolomics in the management of myopic diseases (AUC 0.59–0.98) [14]. However, recent prediction models for metabolomics rely on only a single source of information (differential metabolites) and lack the integrated use of multidimensional information. Machine learning (ML) methods are able to overcome some limitations of conventional risk prediction models, synthesize high-dimensional data from multiple information sources, and deal with high-dimensional, nonlinear and interactive relationships among disease features to achieve data-driven outcome prediction [14,15], and many ML methods have been successfully used for prediction in ophthalmology [2,15,16,17,18,19,20]. Therefore, we sought to develop a ML-based myopic retinopathy prediction model that integrates systematic and metabolic features to predict myopic retinopathy events in children and adolescents.

We hypothesize that myopic retinopathy has a different serum metabolic profile and that ML algorithms can identify disease-specific metabolic patterns that are beyond current ophthalmic knowledge. Our prediction models were developed from a population-based cohort of children and adolescents with myopia, and metabolic features meaningful for discrimination were screened by variable selection. Metabolic features and conventional clinical factors were combined to construct ML, including XGBoost, Support Vector Machine (SVM), and logistic regression, along with parameter estimation. In addition, this prediction model was validated in an independent time-point cohort to assess its generalizability. The results of the study were also compared with the results of previous review studies. This study promotes the formation of an individual-based identification system to predict which patients are at risk of developing myopic retinopathy, which can guide review (close monitoring of disease progression) to avoid progression of vision-impairing events.

## 2. Materials and Methods

### 2.1. Study Design and Participants

This study is part of the large-scale refractive eye development study (September 2020–May 2021) of children and adolescents in Shanghai; detailed cohort descriptions have been reported elsewhere [21]. To recap, the study began in 2018 and is expected to last until 2038. Children and adolescents (aged 4–18 years) in good physical condition are included in the study to establish a refractive developmental profile in Shanghai, and annual follow-ups are planned. Children with organic eye disease, including strabismus, congenital cataract or glaucoma, fundus disease other than myopia-related fundus lesions, and other conditions that interfered with the purpose of the study, such as amblyopia and systemic diseases affecting metabolism, were excluded. All participants and all guardians were informed of the study objectives and study protocol. Written informed consent was obtained from participants over 12 years of age and from all guardians, and verbal informed consent was obtained for children under 12 years of age. The study protocol was conducted in accordance with the Declaration of Helsinki and was approved by the Ethics Committee of Shanghai General Hospital.

### 2.2. Study Protocol

All participants underwent a comprehensive eye examination, questionnaires, and blood sample retention. The study participants and their guardians were required to jointly complete a questionnaire designed specifically for this study, in which the survey included basic information such as sex, age, grade level, and vision-related information. Height and weight were determined with an analogue stadiometer and a digital weight scale, respectively. The ophthalmologic examination included ciliary muscle paralysis optometry, intraocular pressure measurement (NonContact Tonometer, NT-510, Nidek, Tokyo, Japan), and fundus examination after dilatation. After slit lamp examination, one drop of 0.5% proparacaine (Alcaine, Alcon) was placed in each eye, followed by one drop of 1% cyclopentolate (Cyclogyl, Alcon) per eye at 5-min intervals. After an interval of 30 min, eyes with loss of light reflex and pupil diameter larger than 6 mm were considered to have complete cycloplegia. Refraction and corneal curvature were measured after cycloplegia using an automated autorefractor (KR-8900, Topcon, Tokyo, Japan), and the procedure was repeated three times for each eye with refractive differences greater than 0.5 diopter (D). To minimize the effect of diurnal variations, fundus images were acquired between 10:00 and 15:00 daily. Fundus photographs of the macular region were acquired using a digital retinal camera in the Swept-source optical coherence tomography (SS-OCT, DRI OCT Triton, Topcon, Tokyo, Japan) system, with the images focused on the posterior pole including the optic nerve and macula or the peripheral retina. A signal strength ≥ 60 and image quality ≥ 90 was required for each qualified OCT image. Each SS-OCT examination will include 12 radial scan lines focused on the centre of the fovea or the optic disc, for a detailed description.

Fundus photographs were evaluated according to the international photographic classification system (META-analysis for Pathologic Myopia [META-PM] study classification), which classifies macular lesions into five classes: no myopic retinal degenerative lesion (category 0), tessellated fundus only (category 1), diffuse chorioretinal atrophy (category 2), patchy chorioretinal atrophy (category 3), and macular atrophy (category 4) [6]. Unlike myopic retinopathy in adults, retinopathy in children and adolescents is mostly in the primary stage and the degree of lesions is mild. In this study, the presence of category 1 tessellated fundus and above was defined as having myopic retinopathy in children and adolescents according to the META-PM classification system. Two trained ophthalmologists (authors B.Z. and S.L.) independently read the photographs to determine the occurrence of myopic retinopathy. If there was any disagreement, a senior ophthalmologist (authors Xun Xu) was required to make the decision. Spherical equivalent (SE) was calculated as spherical power + 0.5*cylindrical power. Considering that there is a correlation between refraction and axial length, we refer to the criteria recommended by a Japanese study to define myopia: the criteria for HM are a myopic refractive error of more than 6.0 D for children aged 6 to 8 years and more than 8.0 D for children aged 9 years and older [22]. Body mass index was calculated as weight/height^2^ (kg/m^2^).

### 2.3. Metabolite Analysis

Fingertip capillary blood samples were extracted using a 21- or 23-gauge butterfly needle, placed in 4 mL sodium heparin tubes, and immediately stored in a −80 °C cryogenic freezer until analysis. The extraction and chemical derivatization of serum metabolites are briefly described as follows: 100 μL of serum sample was first placed in an EP tube, and then 410 μL of precooled extraction solution (methanol with internal standard 2-chloro-L-phenylalanine) was added and vortexed for 30 s with an aliquot of the sample. The samples were centrifuged at 12,000 rpm (RCF = 13,800 (×*g*), R = 8.6 cm) for 15 min at 4 °C, and 180 μL of supernatant was transferred to a 1.5 mL EP tube. Thirty microliters of each sample was mixed into quality control (QC) samples. The supernatant was completely dried in a vacuum concentrator, and 30 μL of methoxyamination hydrochloride (20 mg/mL in pyridine) was added and incubated at 80 °C for 30 min. Then, 40 μL of BSTFA (1% TMCS, *v/v*) reagent was added to the sample fraction and incubated at 70 °C for 1.5 h. After cooling to room temperature, 5 μL of FAMEs (in chloroform) was added to the QC sample, and finally, the derivatized samples were analysed using a gas chromatograph coupled with a time-of-flight mass spectrometer (GC–TOF–MS).

The GC–TOF–MS analysis was performed using an Agilent 7890 gas chromatograph coupled with a time-of-flight mass spectrometer. The MS-DIAL software [23] and the Fiehn BinBase were used for raw peak exacting, data baseline filtering and calibration of the baseline, peak alignment, deconvolution analysis, peak identification and integration of the peak area [24]. Both mass spectrum matches and retention index matches were considered in metabolite identification. Finally, the peaks detected in less than half of QC samples or relative standard deviation (RSD) > 30% in QC samples were removed.

### 2.4. Statistical and Data Analyses

**Figure 1:**
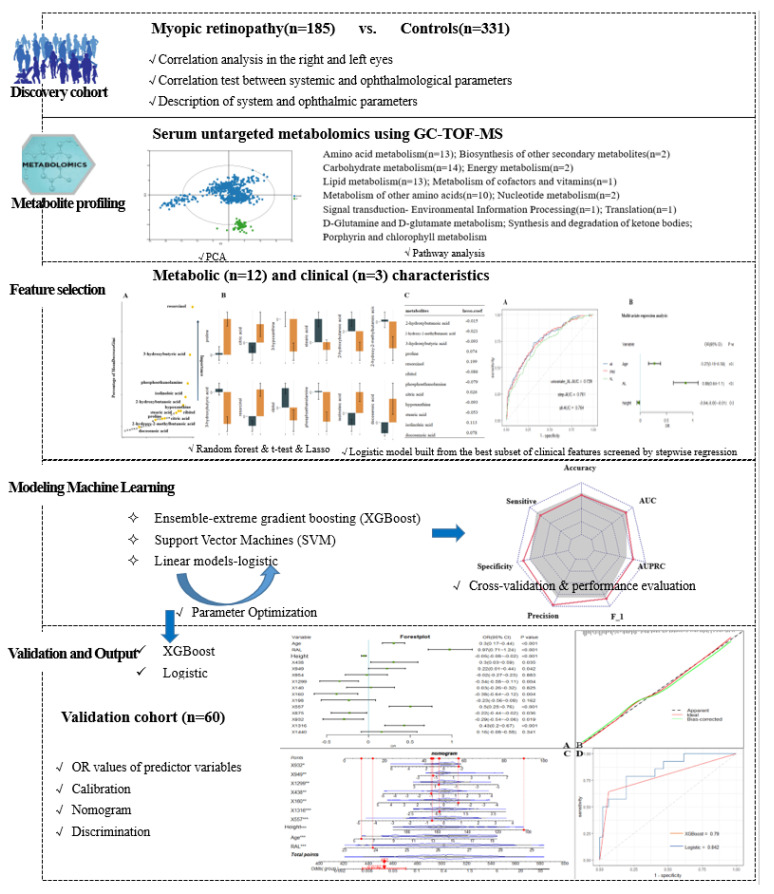
Study workflow and design.

The research design flow of the article is shown in Figure 1. Data collection included demographics, ophthalmic examinations, and metabolic characteristics. Traditional descriptive methods were used to describe the clinical and demographic characteristics, with data described as the mean (SD) or median (interquartile spacing) for continuous variables and as the frequency for categorical variables. The outcome indicator of interest was the presence or absence of myopic retinopathy. For each study characteristic, the Mann–Whitney U test or *t*-test was used for continuous variables, and the chi-square test was used for categorical variables.

In this dataset, 390 named metabolites were identified, and their relative content was expressed in terms of peak intensities. The raw metabolomics data were normalized by sum and auto-scaled to address the large variations and nonnormal distribution of the values. Because the large dimensionality (number of features) of the metabolome data relative to the size of the dataset (number of samples) can cause uncertainty in the location of the separation surface, we used Lasso [25] and RF [26] filtering to study the features before constructing the prediction model. Similar to many studies that attempted to reduce feature dimensionality and increase the robustness of screened features, a combinatorial screening process using features that simultaneously satisfied *p* < 0.05 (*t*-test), ranked in the top 30 of mean decrease in Gini index (DecreaseGini) and had Lasso coefficients not equal to 0 were screened as the final selected metabolic features [27]. Study subjects participating between September 2020 and February 2021 were used as a training cohort for feature screening and ML model construction. For model validation, in addition to cross-validation within the training set, we used a temporally distinct cohort that included subjects participating in the study during March 2021.

We refer to Subudhi et al. [28] for a comparison of ML algorithm applications and select algorithms belonging to three major classes of ensemble, linear, and support vector machines, specifically XGBoost, logistic model, and SVM. Similar to Nezu et al., we performed hyperparameter tuning by using mtry for RF, cost and gamma for SVM, and grid search to obtain the optimal parameters for XGBoost. The performance of the model was evaluated based on a set of learning metrics (accuracy, sensitivity, specificity, precision, F1 score and mean AUC) as a way to illustrate the stable contribution of screened metabolic features to disease identification in different ML models. We also generated receiver operating characteristic curves (ROC) and precision-recall curves (PRC) for the model and calculated the area under those two curves (AUROC and AUPRC, respectively). We used the F1 score and AUPRC as the main performance metrics for model comparison because they are more informative for evaluating binary classifiers on unbalanced datasets. The performance of the filtered metabolic features combined with different ML models was also validated in a temporal validation cohort. Ultimately, to facilitate the application of the model findings, we further evaluated and described the model findings by calculating the predicted and true probabilities for plotting the calibration curves. The closer the calibration curve is to the 45° diagonal, the better the model execution. Nomograms of the study results, which are graphical representations of the predictive statistical models for individual patients, were plotted by scaling each regression coefficient in a multivariate logistic regression to a scale of 0 to 100. The effect of the variable with the highest beta coefficient (absolute value) was assigned a score of 100. The scores of the independent variables are summed to give a total score, which is converted to a predictive probability [29]. Using nomograms, complex predictive models can be reduced to the probability of an event, facilitating clinical translation of study results [30]. Finally, the results of this study were also compared with the results of previous review studies.

## 3. Results

### 3.1. Systemic and Ophthalmological Characteristics

The training cohort was derived from myopic participants who met the recruitment criteria between September 2020 and February 2021, excluding those with low biological sample volumes, haemolysis, and other conditions that affect metabolite detection (*n* = 15), resulting in 516 participants selected for inclusion in the final training analysis. Based on the META-PM study definition, 185 (35.85%) participants in the training cohort had myopic retinopathy, including 153 FT patients and 32 diffuse chorioretinal atrophy. Thirteen (21.67%) of the 60 participants in the temporal validation dataset had myopic retinopathy, including 12 patients with FT and one patient with diffuse chorioretinal atrophy. The systemic and ophthalmic characteristics of the study cohort are shown in Table 1. Participants in the validation cohort were younger than those in the training cohort and had a higher spherical power (SE). In the study cohort, the age was 14.55 (12.13, 16.52) years, and 267 (51.74%) were girls. The mean AL was 26.21 ± 1.15 mm; the SE was −6.00 (−7.00, −5.25); the cylindrical power was −1.25 (−2.25, −0.75); the mean radius of curvature was 7.77 ± 0.26; and the pupil distance was 60.00 (57.00, 63.00). There were 135 (26.16%) subjects with HM among all participants. Three parameters, anterior chamber depth, central corneal thickness, and lens thickness, were missing in greater than 15% of cases and were not included in the follow-up analysis.

In the training cohort, the correlation test between the systemic and ocular parameters of the right and left eyes of the patients showed that the correlation coefficients of all characteristics were greater than 0.73, except for SE (r = 0.665), and all correlation coefficients were statistically significant (*p* < 0.05). Therefore, in this study, only the ophthalmic parameters of the right eye of the study subjects were used for analysis. Among participants with or without retinopathy, the systematic parameters of age, height, weight, and BMI, and the ophthalmic parameters of AL, SE, mean radius of curvature, and pupil distance were significantly different (*p* < 0.05), but anterior chamber depth, central corneal thickness, lens thickness, SE, and sex did not differ. Retinopathy was proportionally higher in participants with HM than in those with mild to moderate myopia. Retinopathy was still present in 109 (28.61%) of the 381 participants with mild to moderate myopia (Table 2).

The correlation test between each characteristic parameter (Supplementary Figure S1) showed that the mean radius of curvature did not have statistically significant correlation coefficients with age, SM or SE. AL was negatively correlated with SE and SM with correlation coefficients > 0.6; weight was positively correlated with age and height with correlation coefficients > 0.7.

### 3.2. Systemic and Ophthalmological Parameters for Classifying Retinopathy in Training Cohort

Univariate logistic models for the occurrence of retinopathy were constructed by selecting all baseline characteristics separately. The results showed that the univariate AUC ranged from 0.544 to 0.729; only the AUC of the AL model was 0.729 (CI: 0.684–0.774), and the rest of the models were less than 0.7. Referring to the results of the correlation analysis among features, a total of seven baseline features were selected to construct multivariate logistic models, and an AUC of 0.764 (CI: 0.721–0.806) was obtained for all the variables. The optimal subset of age, AL, and height, which were screened by stepwise regression, obtained an AUC of 0.761 (CI: 0.718–0.804). After running the R function roc.test, which performs the area under the ROC curve difference test, it was shown that the multivariate model differed from the univariate model, and there was no difference between the two multivariate models. The final modelling results are shown in Supplementary Figure S2.

### 3.3. Identification of Serum Nontargeted Metabolic Profiles and Metabolic Signatures in Myopic Retinopathy

**Figure 2:**
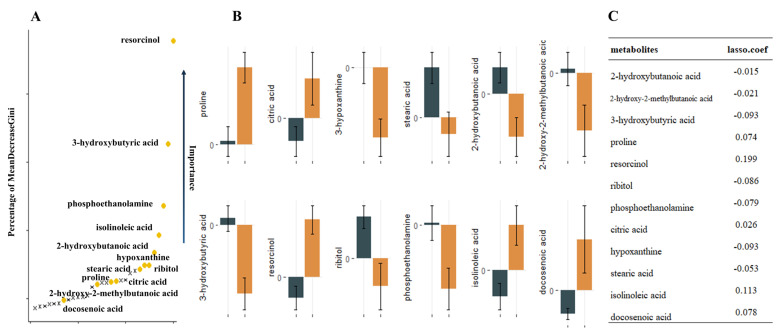
12 metabolites extracted by the three methods repeatedly. (**A**). Illustration of a random forest graph. The graph shows the top thirty metabolites, the vertical axis shows the proportion of metabolite Gini coefficient reduction among all metabolites, and the metabolites extracted repeatedly with Lasso and *t*-test are marked in yellow. (**B**). Error bars of metabolite relative concentrations. (**C**). Correspondence coefficients of Lasso extraction features.

Nontargeted metabolomics analysis identified 390 named metabolites. Unsupervised model principal component analysis of all samples showed tight aggregation of QC samples (Figure 1) and a peak area RSD ≤ 30% for internal standard substances in QC samples, supporting the robustness of the metabolic assay platform operation. Metabolites were matched to 319 HMDB numbers present in 62 metabolic pathways, mainly involving amino acid metabolism and carbon dioxide metabolism-related pathways. Using univariate *t*-test analysis, we observed significant between-group variation in quantity, corresponding to a decrease, for 27 metabolites: 2-hydroxybutanoic acid, 2-hydroxy-2-methylbutanoic acid, 3-hydroxybutyric acid, ribitol, phosphoethanolamine, hypoxanthine, stearic acid, homoserine, linoleic acid, glycolic acid, maleimide, glycerol, and N-carbamoylaspartate. In addition, we found that proline, resorcinol, citric acid, isolinoleic acid, docosenoic acid, valine, oxamic acid, isothreonic acid 1, methyltetrahydrophenanthrenone 2, citrulline, pinitol, histidine, monomyristin, and 1-monoheptadecanoyl glyceride were significantly increased. Sixteen metabolic features remained after Lasso model screening that were relevant for classification. Finally, the 12 metabolites that were repeatedly extracted by the three methods (*t*-test, Lasso and RF) were obtained for the next model construction by combining the metabolic features ranked in the top 30 of the Gini index reduction (DecreaseGini) in the RF model (Figure 2).

### 3.4. Machine Learning Disease Prediction Model Based on Metabolic Features and Clinical Data

**Figure 3:**
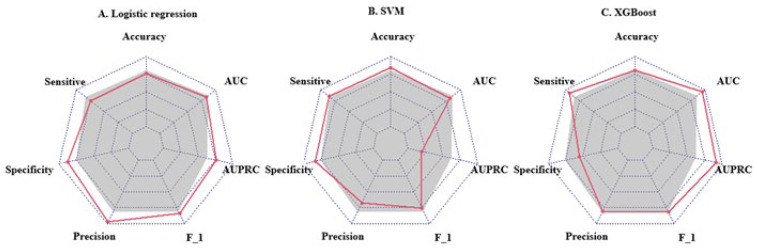
Radar plot of evaluation metrics for machine learning prediction models. The axes starting from the same starting point indicate multiple evaluation metrics with a starting point of 0 and an end point of 1. The points on the corresponding axes of the evaluation metrics indicate the size of the metric, and the grey shading corresponds to the mean value of the three models. F_1 = F1 score; AUC = area under receiver operating characteristic curve; AUPRC = area under the precision-recall curve.

**Figure 4:**
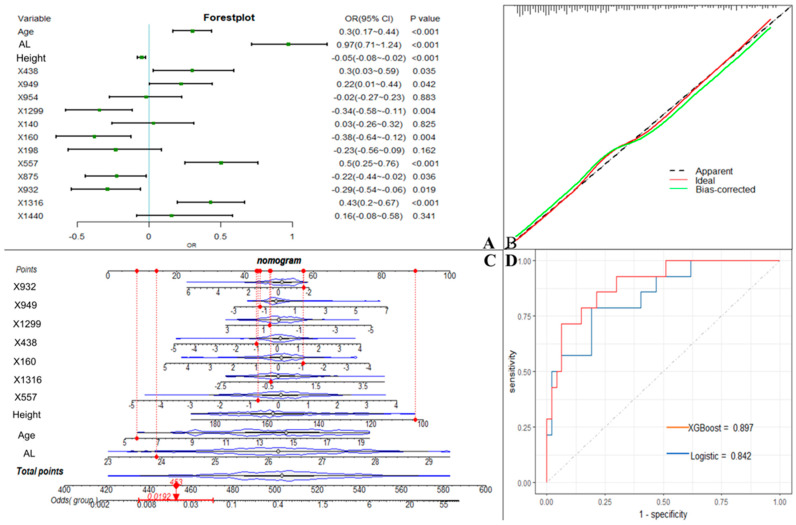
Machine learning model incorporating metabolic features. (**A**). OR values of predictor variables and their 95% confidence intervals in the logistic model incorporating metabolic features. (**B**). Calibration curves of logistic model column line graphs in the validation cohort. (**C**). Estimation of the probability of macular lesions by creating column line plots in the training set. (**D**). Area under the receiver operating characteristic curve for predicting the sensitivity and specificity of the model in the validation set, as determined by 2 machine learning algorithms: support vector machine and logistic regression. X438 (proline), X949 (citric acid), X954 (hypoxanthine), X1299 (stearic acid), X140 (2-hydroxybutanoic acid), X160 (2-hydroxy-2-methylbutanoic acid), X198 (3-hydroxybutyric acid), X557 (resorcinol), X875 (ribitol), X932 (phosphoethanolamine), X1316 (isolinoleic acid), X1440 (docosenoic acid).

We used the screened 12 metabolic features and three clinical features for the construction of ML models. Figure 3 summarizes the accuracy, sensitivity, specificity, precision, F1 scores, AUROCs, and AUPRCs for the three ML prediction models. We observed that the AUCs for all models in the training cohort were greater than 0.8. Specifically, the AUCs were 0.833 (95% CI, 0.797–0.869), 0.810 (95% CI, 0.774–0.846), and 0.950 (95% CI, 0.933–0.966) for the logistic regression, SVM, and XGBoost classifiers, respectively. The F1 scores of both the logistic model and XGBoost were greater than 0.8, and the AUPRC was greater than 0.75. To facilitate the application of the model findings, we selected the logistic model and XGBoost with better F1 scores and AUPRC performance for further evaluation and description. The 10-fold cross-validation of the logistic model in the training cohort showed AUC = 0.811. In the validation cohort (Figure 4D), the logistic model yielded 0.842 (95% CI, 0.724–0.96), and the XGBoost model showed an AUC of 0.897 (95% CI, 0.807–0.986). Three clinical characteristic coefficients remained significant in the multivariate joint model (*p* < 0.05), as did 8/12 metabolic characteristic coefficients (Figure 4A). Based on the results of the multivariate logistic regression, a column line plot (Figure 4C) was developed and presented to validate the results, showing a good calibration curve for the constructed model (Figure 4B).

### 3.5. Comparison of the Focused Metabolic Profiles with Those of the Myopic Population

Of the overlapping metabolites screened by the three methods, four metabolites (4/12) were identified in previous myopia-related metabolomics studies, with citric acid, proline, and hypoxanthine showing the same direction of change as the case group in this study (Table 3). In the study by Ke et al., serum citric acid levels increased between HM and mild to moderate myopia in Chinese elderly individuals, and the AUC value to distinguish HM from mild myopia was 0.69. Hypoxanthine and stearic acid were selected as potential serum biomarkers to distinguish pathological myopia in the study by Liu et al., but hypoxanthine exhibited the opposite direction of content change from the present study. In an animal vitreous and retinal metabolomics study in which the spectral content of ambient white light affected eye growth, FDEP eyes had lower levels of proline than controls under SW light, where proline is often present as an energy substrate in the retina.

## 4. Discussion

We performed a novel serum metabolomics study to systematically characterize the metabolic profile of the primary stages of myopic retinopathy in children and adolescents. We further used multiple feature screening methods to identify 12 metabolites important for the diagnosis of myopic retinopathy, most of which were associated with energy metabolism. A combination of 12 metabolites and clinical parameters was constructed using three ML algorithms to distinguish myopic retinopathy from myopic participants. Two models with better performance were validated in a time-validated cohort, and both showed good prediction results.

Myopic retinopathy is the second most common cause of blindness in China and the leading cause of blindness in Shanghai [31]. Previous studies have focused on HM [32,33,34], but there is growing evidence that the prevalence of fundus abnormalities in children with mild to moderate myopia is much higher than expected [35,36], and this study found that myopic retinopathy was present in 28.61% of children with mild to moderate myopia. Long-term longitudinal observational studies have shown that childhood FT and diffuse chorioretinal atrophy readily progress to pathological myopia in adulthood and may be an earlier marker for the development of late-onset myopic retinopathy [37,38,39,40]. Yokoi et al. showed that 83% (35 eyes) of eyes with pathologic myopia in adulthood already had diffuse chorioretinal atrophy in childhood [41]. In the population-based Beijing Eye Study, after 10 years of follow-up, 19% (15 eyes) of baseline FT developed myopic maculopathy at the end of follow-up [42]. Fundus tessellation has also shown good predictive value for myopic maculopathy in the Singapore Longitudinal Study of Adult Myopia over 12 years [4]. Some studies have shown that myopia presents with reduced retinal and choroidal blood perfusion and that FT is accompanied by choroidal capillary atrophy [43]. Therefore, the identification and study of early mild fundus changes in children and adolescents are essential to prevent later visual impairment. Higher age and higher myopic refraction are the main risk factors, and other risk factors include longer axis length and male sex [4,36,44,45]. A risk factor analysis of FT in junior high school students conducted in Beijing showed that high-grade FT was associated with reduced subfoveal choroidal thickness and longer AL [46]. The present study also found that the group with fundus changes was older, had longer axial length and had higher myopia than the group of myopic children and adolescents without fundus changes. However, tests for differences in spherical power and cylindrical power specifically showed that cylindrical power did not differ between myopic children and adolescents with or without fundus changes. Previous studies have shown that higher grades of FT are independently associated with greater corneal radius (CR) and that the extent and proportion of FT increases dramatically with increasing CR [35]. In children with low myopia and high CR, FT may have been present for a long time if the compensatory effect of the cornea was not coordinated with the growth of AL, and if the progression of myopia began to accelerate. Therefore, combining the correlation studies between systemic and ophthalmic parameters, we included seven features in a multivariate logistic model, and the optimal subset of the remaining three features that were screened by stepwise regression, age, AL, and height, were included in the next ML model, and combined with metabolic features.

This was the first study to focus on the early stages of myopic retinopathy in children and adolescents, identifying differences in serum metabolic patterns to further enable accurate prediction of myopic retinopathy (FT and diffuse chorioretinal atrophy). A nontargeted metabolomics approach detected 390 named metabolites present in 62 metabolic pathways, mainly involving amino acid (*n* = 13), carbohydrate (*n* = 14), and lipid (*n* = 13) metabolism-related pathways. Using univariate *t*-test analysis, we observed 27 metabolites that varied significantly in abundance between groups. Predictors were further screened from those 27 metabolites to construct parsimonious models that could be more easily implemented in clinical settings. Pathway analysis revealed that differential metabolites in pathways related to carbohydrate metabolism (galactose metabolism; citrate cycle), amino acid metabolism (arginine biosynthesis; alanine, aspartate, and glutamate metabolism) and translation (aminoacyl-tRNA biosynthesis) were meaningfully enriched (*p* < 0.05). The existence of complex interactions between metabolites or the presence of interrelated metabolic pathways makes it difficult to screen for stable predictors by traditional feature selection methods, such as logistic regression. This study further utilizes Lasso and RF algorithms to filter features. Lasso reduces multicollinearity of metabolic features and retains metabolites with nonzero coefficients [47]. The RF algorithm is a tree-structure-based method that determines the most important variables in the classification after taking into account the complex nonlinear relationships in the dataset. Twelve metabolic features, six with rising and six with falling metabolite expression levels, were finally selected for multivariate modelling. Proline, citric acid, and hypoxanthine all affect retinal energy metabolism. Proline was increased in the vitreous of control and recovered FDEP eyes under BEW light [48]. Proline, a major nutrient for RPE cells, mediates the metabolic exchange between RPE cells and the retina and is often reported to influence AMD development in metabolomics studies [49,50]. Citric acid was found to be increased in serum in both the present study and in the study by Ke et al. [51]. Citric acid is an important intermediate in energy metabolism, and changes in energy metabolism affect the expression of extracellular adenosine receptors, which affect growth regulation in the eye [52]. Studies of 7-methylxanthine associated with myopic children have shown that energy metabolism indirectly affects the rate of eye axis elongation and myopia progression [53]. Similarly, hypoxanthine is a reactive intermediate in adenosine metabolism and nucleotide salvage pathways to form nucleic acids, which mainly affect the purine nucleotide cycle. Purines are not only involved in intracellular energy metabolism but also activate intercellular communication through receptors, leading to photoreceptor and RPE cell apoptosis [54,55]. Upon deeper study, we also found that 3-hydroxybutyric acid is involved in the synthesis and degradation of ketone bodies and butanoate metabolism in two pathways. Citrate similarly affects the citrate cycle and alanine. This suggests that there are interactions between disease pathways in myopic retinopathy and that future treatments may need to target multiple pathways simultaneously.

We trained metabolite and clinical features using a selection of three ML algorithms belonging to the more classical of the three classes of ensemble, linear, and SVM algorithms, and the results showed that the logistic and XGBoost-based models were the better performing models, with high accuracy of high discrimination in both the internal cross-validation and external time-validation cohorts. When the size of the study classes is very different, such as in this study, where the number of control and case groups was 331:185 and there were significantly more study subjects in the control group than in the case group, the standard classification algorithm may favour large classes, resulting in poor accuracy of minority class predictions [56,57]. Therefore, the evaluation criteria of the algorithms used in this study also differ from previous studies that focused only on the sensitivity and specificity of classification algorithms but used F1 scores and AUPRC as the main performance indicators for model comparison because they are more informative for evaluating binary classifiers on unbalanced datasets.

An important advantage of this study is that it was prospectively designed, and all data collection was standardized according to a predetermined protocol. In addition, participants underwent a comprehensive ophthalmologic examination performed by a retinal specialist, ensuring excellent phenotypic characteristics. Our study was the first to develop an ML-based classifier of serum metabolites and clinical features to predict the occurrence of myopic fundus lesions, and rigorous steps were used for model specification and model performance assessment (i.e., discrimination, calibration, and clinical utility). However, our study also has limitations. First, because the current grading system is based on data from the adult population, strict criteria for fundus lesions may lead to the omission of younger patients. The same lack of recent studies on myopic retinopathy in children and adolescents makes our findings lack reference and comparison. However, under limited conditions, the present study compared the identified focal metabolites with potential biomarkers summarized by previous myopia metabolomics review studies. Second, vitreous, atrial fluid, and other ocular tissue samples are difficult to obtain in myopia-only studies. Blood biomarkers are less invasive, more accessible, and easier to obtain, but studies are currently limited to order-of-magnitude characteristic associations, and we have not been able to elucidate the specific effects of blood metabolites on myopia. More importantly, the cross-sectional design does not allow us to investigate the timeline pattern between risk factor exposure and its effect on the disease. Some relationships may reveal consequences of ocular primary disease rather than risk factors. Changes in the levels of some metabolites may indicate pathophysiological pathways of disease (thus occurring prior to disease onset) or consequences of disease or systemic drug therapy (subsequently occurring after disease onset). Furthermore, this study represents only a snapshot of the metabolome of the participants studied. However, the metabolome is highly dynamic and vulnerable to external factors. Longitudinal studies are needed to confirm our findings and to assess the evolution of the metabolome with the progression of myopic retinopathy. To reduce the impact of false-positive results, our findings were well discriminated in multiple ML model validation cohorts, yet further refinement of the study is still needed, such as experimental studies to reveal the exact mechanism of screening out metabolites. Finally, XGBoost-based prediction models may still be difficult to interpret compared to regression models that simply use given coefficients to weight predictors, so logistic and XGBoost models were selected for further analysis in this study. Overall, the obtained findings contribute to a better understanding of the pathogenesis of myopic retinal changes and potentially support decisions regarding the method design of future ML classifiers. Future work should address these limitations by linking these findings to genetic risk profiles of patients and controls, which may provide important insights into the pathogenesis of the disease.

## 5. Conclusions

In summary, this hypothesis-free metabolomics study confirmed the existence of a different metabolic profile between myopic retinopathy and controls in children and adolescents. Using a series of ML algorithms, we developed a combined parameter of serum metabolites and clinical features for predicting the onset of the primary stage of myopic retinopathy in children and adolescents. Our study complements the results of myopic retinopathy studies in children and adolescents and contributes to the development of precision medicine for myopic retinopathy.

## Figures and Tables

**Table 1:** Systemic and ophthalmological parameters of included cohorts.

Characteristic	%Missing	Training Set (*n* = 516)	Validation Set (*n* = 60)	%Missing
Age, years		14.20 ± 3.07	12.95 ± 3.05	
height, cm		155.20 ± 16.20	151.03 ± 19.91	
weight, kg		50.00 (39.77, 60.40)	48.30 (31.73, 56.23)	
Body mass index, kg/ $m^{2}$		19.70 (17.73, 22.64)	19.70 (17.53, 22.11)	
Axial length, mm		26.21 ± 1.15	25.60 (25.12, 26.73)	
Anterior chamber depth, mm	18.99	3.80 ± 0.22	3.81 ± 0.23	
Central corneal thickness, um	18.80	540.00 (518.00, 563.50)	536.90 ± 35.37	
Lens thickness, mm	22.29	3.37 (3.28, 3.48)	3.34 ± 0.16	40.00
spherical power, diopters		−6.00 (−7.00, −5.25)	−5.25 (−6.31, −3.94)	
cylindrical power, diopters		−1.25 (−2.25, −0.75)	−0.75 (−1.50, −0.50)	
Spherical equivalent, diopters		−6.88 (−8.13, −5.88)	−6.06 (−7.25, −4.34)	
Mean radius of curvature, mm		7.77 ± 0.26	7.80 ± 0.25	
Pupil distance, mm		60.00 (57.00, 63.00)	60.10 ± 4.27	
Female (%)		267 (51.74)	29 (48.33)	
Macular lesions (%)		185 (35.85)	13 (21.67)	
Fundus tessellation (FT) (%)		153 (29.88)	12 (20.00)	
Diffuse chorioretinal atrophy (%)		32 (0.63)	1 (1.67)	
High myopia (%)		135 (26.16)	12 (20.00)	

**Table 2:** Comparison of Systemic and ophthalmological parameters of Subjects with and without macular lesions.

Characteristic	Controls (*n* = 331)	Myopic Retinopathy (*n* = 185)	H/t	*p*
Age, years	13.34 (11.62, 15.78)	15.97 (13.63, 17.65)	47.990	<0.001
height, cm	155.00 (142.00, 163.00)	162.25 (154.00, 170.50)	33.600	<0.001
weight, kg	46.40 (35.45, 57.20)	53.80 (47.10, 64.10)	31.128	<0.001
Body mass index, kg/ $m^{2}$	19.14 (17.22, 22.38)	20.26 (18.73, 23.26)	15.349	<0.001
Axial length, mm	25.87 ± 1.03	26.82 ± 1.10	−9.567	<0.001
Anterior chamber depth, mm	3.81 ± 0.21	3.80 ± 0.24	0.310	0.757
Central corneal thickness	538.50 (518.00, 563.00)	543.00 (519.00, 564.00)	0.660	0.417
Lens thickness, mm	3.36 (3.28, 3.47)	3.38 (3.28, 3.50)	0.484	0.487
spherical power, diopters	−5.75 (−6.50, −4.75)	−6.75 (−8.00, −5.75)	55.802	<0.001
cylindrical power, diopters	−1.25 (−2.25, −0.75)	−1.50 (−2.25, −1.00)	2.824	0.093
Spherical equivalent, diopters	−6.50 (−7.56, −5.50)	−7.63 (−8.88, −6.50)	47.867	<0.001
Mean radius of curvature, mm	7.73 ± 0.24	7.83 ± 0.26	−4.240	<0.001
Pupil distance, mm	59.00 (56.00, 62.00)	61.00 (58.00, 63.00)	22.035	<0.001
			${ꭓ}^{2}$	
Female%	179 (54.08)	88 (47.57)	1.763	0.184
Male%	152 (45.92)	97 (52.43)		
Mild to moderate myopia%	272 (71.39)	109 (28.61)	32.033	<0.001
High myopia%	59 (43.70)	76 (56.30)		

**Table 3:** Comparison with overlapping metabolites in myopic population studies.

Metabolite	Citric Acid ↑	Proline ↑	Hypoxanthine ↓	Stearic Acid ↓
Hit	Ke (2020)	Najjar (2021)	Liu (2020)	Liu (2020)
Case	40 HM	18 chicks with monocular FD (BEW light)		57 PM
Control	40 mild myopes	18 chicks with monocular FD (3900 K SW LED light)		81 cataract patients
Age (case)	≥60	-		55.32 ± 14.49
Age (control)	match	-		65.83 ± 11.94
Patients vs. Controls	Increased	Increased	Decreased	Increased
Biofluid	Serum	vitreous and retinas	Serum	Serum
Technique Employed	GC-TOF-MS	LC-MS		GC-TOF-MS
Evaluation standard	PLS-DA (VIP > 1.0) and *t*-test (*p* < 0.05)	PLS-DA and OPLS-DA		OPLS-DA (VIP > 1.0), *t*-test (*p* < 0.05), and FC > 1.2 or <0.8

GC = gas chromatography, TOF = time of flight, MS = mass spectrometry; PLS-DA = Partial least squares discriminant analysis, FC = fold change, VIP= variable importance projection; PM = pathological myopia, HM = high myopia, FD = form deprivation.

## Data Availability

The dataset supporting the conclusions of this paper can be obtained by sending an email request to the corresponding author. The data are not publicly available due to privacy.

## Supplementary Materials

The following are available online at https://www.mdpi.com/article/10.3390/metabo13020301/s1, Figure S1: The correlation test between each characteristic parameter, Figure S2: After running the R function roc. test, which performs the area under the ROC curve difference test, it was shown that the multivariate model differed from the univariate model, and there was no difference between the two multivariate models.
