# Supplementary figures and images for: Machine Learning-Based Integration of Metabolomics Characterisation Predicts Progression of Myopic Retinopathy in Children and Adolescents
Source: Metabolites. 2023 Feb 17;13(2):301. doi: 10.3390/metabo13020301 (PMC9965721; doi:10.3390/metabo13020301)

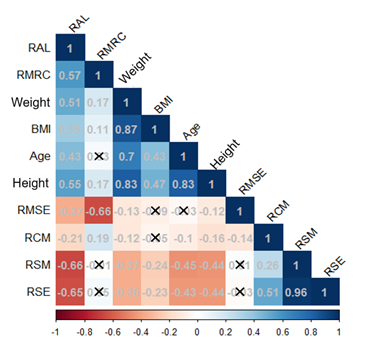

Supplement: Supplementary file 1 [file metabolites-13-00301-s001.zip › Supplementary Figure S1..tiff]

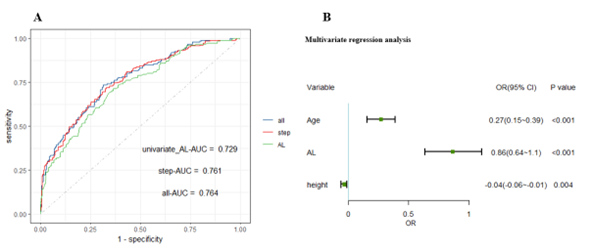

Supplement: Supplementary file 1 [file metabolites-13-00301-s001.zip › Supplementary Figure S2.tiff]
